# Adiponectin Is a Contributing Factor of Low Appendicular Lean Mass in Older Community-Dwelling Women: A Cross-Sectional Study

**DOI:** 10.3390/jcm11237175

**Published:** 2022-12-02

**Authors:** Leonardo Augusto Costa Teixeira, Jousielle Marcia dos Santos, Adriana Netto Parentoni, Liliana Pereira Lima, Tamiris Campos Duarte, Franciane Pereira Brant, Camila Danielle Cunha Neves, Fabiana Souza Máximo Pereira, Núbia Carelli Pereira Avelar, Ana Lucia Danielewicz, Amanda Aparecida Oliveira Leopoldino, Sabrina Paula Costa, Arthur Nascimento Arrieiro, Luana Aparecida Soares, Ana Caroline Negreiros Prates, Juliana Nogueira Pontes Nobre, Alessandra de Carvalho Bastone, Vinicius Cunha de Oliveira, Murilo Xavier Oliveira, Pedro Henrique Scheidt Figueiredo, Henrique Silveira Costa, Vanessa Amaral Mendonça, Redha Taiar, Ana Cristina Rodrigues Lacerda

**Affiliations:** 1 Physiotherapy Department, Federal University of Jequitinhonha and Mucuri Valleys, Diamantina 39803-371, Brazil; 2 Postgraduate Program in Health Sciences (PPGCS), Federal University of Jequitinhonha and Mucuri Valleys (UFVJM), Diamantina 39803-371, Brazil; 3 Postgraduate Program in Rehabilitation and Functional Performance (PPGReab), Federal University of Jequitinhonha and Mucuri Valleys, Diamantina 39803-371, Brazil; 4 Postgraduate Multicenter Program in Physiological Sciences (PPGCF), Federal University of Jequitinhonha and Mucuri Valleys (UFVJM), Diamantina 39803-371, Brazil; 5 Medicine School, University of Jequitinhonha and Mucuri Valleys (UFVJM), Diamantina 39803-371, Brazil; 6 Department of Health Sciences, University of Santa Catarina (UFSC), Araranguá 88040-900, Brazil; 7 Postgraduate Program in Health Sciences, Faculty of Medical Science of Minas (FCMMG), Belo Horizonte 30130-110, Brazil; 8 MATériaux et Ingénierie Mécanique (MATIM), Université de Reims Champagne-Ardenne, 51100 Reims, France

**Keywords:** aging, appendicular muscle mass, inflammation, biomarkers, adiponectin, sarcopenia

## Abstract

Inflammation is a chronic, sterile, low-grade inflammation that develops with advanced age in the absence of overt infection and may contribute to the pathophysiology of sarcopenia, a progressive and generalized skeletal muscle disorder. Furthermore, a series of biomarkers linked to sarcopenia occurrence have emerged. To aid diagnostic and treatment strategies for low muscle mass in sarcopenia and other related conditions, the objective of this work was to investigate potential biomarkers associated with appendicular lean mass in community-dwelling older women. This is a cross-sectional study with 71 older women (75 ± 7 years). Dual-energy X-ray absorptiometry was used to assess body composition. Plasmatic blood levels of adipokines (i.e., adiponectin, leptin, and resistin), tumor necrosis factor (TNF) and soluble receptors (sTNFr1 and sTNFr2), interferon (INF), brain-derived neurotrophic factor (BDNF), and interleukins (IL-2, IL-4, IL-5, IL-6, IL-8, and IL-10) were determined by enzyme-linked immunosorbent assay. Older women with low muscle mass showed higher plasma levels of adiponectin, sTNFr1, and IL-8 compared to the regular muscle mass group. In addition, higher adiponectin plasma levels explained 14% of the lower appendicular lean mass. High adiponectin plasmatic blood levels can contribute to lower appendicular lean mass in older, community-dwelling women.

## 1. Introduction

According to the International Classification of Diseases (ICD-10-MC), sarcopenia is a gradual and generalized muscle disease that consists of the progressive loss of muscle mass, muscle strength, and/or physical function [1]. Older people with loss of muscle mass experience a marked decline in strength, and physical function [2]. Low muscle mass and function may result in a reduced quality of life, a loss of independence, a need for long-term care, an increased risk of falls and fractures, and mortality [1,2]. Cognitive impairment, fear of falling, depressive symptoms, a poor or fair self-perception of health, and inflammation are a few of the characteristics that might predispose a person to sarcopenia [3].

A continuous, sterile, low-grade inflammation known as “inflammaging” may contribute to the clinical symptoms of chronic diseases [4,5,6]. Thus, inflammaging can contribute to the etiology of sarcopenia and to the dysfunction of skeletal muscle tissue, establishing a vicious cycle of inflammation and muscle wasting [5]. High levels of interleukin-6 (IL-6), interleukin-8 (IL-8), tumor necrosis factor (TNF), interferon (IFN), granulocyte stimulating factor, high-temperature monocytes, and serine protease are frequently associated with deteriorated physical function, reduced muscle mass, and decreased muscular strength in older individuals [7].

Moreover, previous research has demonstrated a negative correlation between inflammatory biomarkers and chronic conditions [6]. In older people with sarcopenia, high levels of IL-6 and soluble tumor necrosis factor receptor 2 (sTNFr2) were reported [4]. Additionally, sarcopenia can affect the signaling pathway for adiponectin activation [8] to prevent muscle atrophy and inflammation while fostering muscle regeneration [8,9,10]. Despite the current literature indicating a link between biomarkers and sarcopenia, especially in women [8], few studies have used DXA to assess appendicular lean mass (ALM). With this regard, current literature often estimates lean mass indirectly or infers muscle mass using a muscular performance test. Since muscle and adipose tissue are related to the release of adipocytokines, it is imperative to assess them using the most recommended method [7,8,9,10]. Furthermore, few studies provide information on the association between muscle mass and a large panel of biomarkers analyzed simultaneously.

It is challenging to identify a single biomarker that can characterize sarcopenia due to the complex pathophysiology and the multiple pathways that cause muscle wasting and sarcopenia [4,8]. Therefore, the aims of the present study were: (1) To classify older community-dwelling women according to the ALM using dual-energy X-ray absorptiometry (DXA), one of the most recommended methods to assess the body composition, as with normal or low ALM. (2) To compare anthropometric parameters, muscle strength, and a broad panel of biomarkers (IL-2, IL-4, IL-5, IL-6, IL-8, IL-10, TNF, sTNFr1, sTNFr2, adiponectin, leptin, resistin, and BDNF) between older community-dwelling women groups stratified according to the ALM. (3) To investigate contributing factors to low ALM in older community-dwelling women. (4) To contribute to the development of strategies for early diagnosis and/or treatment of low muscle mass in sarcopenia and other conditions affected by muscle mass in older women. 

## 2. Materials and Methods

### 2.1. Design

This cross-sectional study was approved by the Ethics Committee of the Universidade Federal dos Vales do Jequitinhonha e Mucuri (UFVJM) with number 1.461.306. All participants signed an informed consent form. The assessments were performed in the Laboratório de Fisiologia do Exercício (LAFIEX) and Laboratório de Inflamação e Metabolismo (LIM) of the UFVJM between June 2016 and June 2017.

### 2.2. Sample

To identify older women compatible with the assessment procedures, community-dwelling older women were recruited based on their registration at the Basic Health Units (BHU) in Diamantina, Minas Gerais, Brazil. All participants were visited at home and answered a questionnaire with information about their medical history, life habits, and comorbidities. The exclusion criteria were age (<65), cognitive decline evaluated by the Mini Mental State Examination [3], subjects unable to walk independently, hospitalized or had suffered fractures in the last three months, presence of neoplasm in the last five years, in palliative care, subjects that performed physical activity on a regular basis (more than three times a week), and subjects with severe visual and auditory impairment and with acute cardiorespiratory diseases. 

### 2.3. Procedures

Three planned sessions included assessments. At first, they completed the clinical health interviews after signing the permission form, and the eligibility criteria were checked. On the newly scheduled day, the participants had examinations of their body composition in the morning while abstaining from food, liquids, and medicine. After a 15-min pause for rest and a standard meal, all participants started the handgrip strength test. To describe the inflammatory profile, blood samples were taken from the participants twenty-four hours after body composition [11].

### 2.4. Biomarker Assessment

Blood was drawn from the patient’s upper limb through antecubital venipuncture and preserved in 10-mL heparin tubes. After collecting blood, the samples were centrifuged at 3000 rpm in a centrifuge for 10 min. Plasma samples were stored in the freezer −80 °C until the analysis. After six months of storage, the inflammatory blood profile was evaluated by measuring the plasma levels of biomarkers IL-2, IL-4, IL-5, IL-6, IL-8, IL-10, IFN, TNF, adiponectin, leptin, resistin, BDNF, and soluble receptors sTNFR1 and sTNFr2 by an immunoenzymatic technique (ELISA sandwich) (DuoSet, R&D Systems, Minneapolis, MN, USA) according to the manufacturer’s instructions [11].

### 2.5. Muscle Stregth

Handgrip strength (HGS) was evaluated using a Jamar dynamometer^®^. The participants were instructed to stay seated, with a neutral fist, an elbow flexed 90 degrees, and a neutral shoulder. The HGS measure, i.e., an isometric contraction of the dominant hand applied on the handles of the dynamometer, was expressed in kilogram force (kgf). The mean of three measurements was used for analysis [2,12].

### 2.6. Body Composition

Body composition was measured using DXA with Encore Software 2005 (Lunar Radiation Corporation, Madison, WI, USA, model DPX). The same assessor performed all body composition evaluations in the morning. After screening by DXA at the ALM, total fat mass and total trunk mass were obtained.

### 2.7. Dependent Variable—Appendicular Muscle Mass 

From the sum of the lean muscle masses of the arms and legs obtained by DXA, it was possible to determine the ALM [2,13]. According to the Foundation for the National Institutes of Health Sarcopenia Project (FNIH) [13] and European Working Group on Sarcopenia in Older People (EWGSOP 2018) [2] the criteria for the low appendicular muscle mass was less than 15.00 kg. The participants were classified into two groups: one with normal ALM and the other with low ALM.

### 2.8. Independent Variables 

The following sixteen independent variables (total fat mass, trunk fat mass and biomarkers IL-2, IL-4, IL-5, IL-6, IL-8, IL-10, IFN, TNF, adiponectin, leptin, resistin, BDNF, sTNFR1, and sTNFr2) were included as possible independent factors associated with ALM.

### 2.9. Analyses

GPower software version 3.1.9.2, was used to calculate the sample size. We estimated the sample size from previous work, which found a correlation between adiponectin and sarcopenia of 0.24 and an effect size of 0.31 [14]. In addition, considering an alpha error of 5% and a power of 80%, a sample size of 71 old people was determined.

The Statistical Package for the Social Sciences, version 22.0 (SPSS Statistics; IBM, Armonk, NY, USA), and MedCalc Statistical Software, version 13.1 (MedCalc Software, Ostend, Belgium), were used to conduct statistical analyses. Data normality was verified by the Kolmogorov–Smirnov test. Continuous variables were expressed as the mean and standard deviation (± SD). The *t*-test for parametric variables or Mann–Whitney-test for nonparametric variables were used to compare the mean differences of continuous variables among subjects with and without low ALM. Pearson or Spearman correlations analysis was used to investigate the relationship between ALM and anthropometric variables and biomarkers. 

Univariate and stepwise multivariate linear regression were used to confirm the determinants of ALM. In each multivariate model adjusted for age, variables related to ALM in the univariate analysis (*p* < 0.1) were included. Four assumptions were used in the linear regression analysis: linearity, residual distribution, homoscedasticity, and the absence of multicollinearity. Scatter plots were used to assess the linearity of the independent variables and residuals, and a histogram was used to look at the distribution of residuals. The scatter plot confirmed the homoscedasticity, which was defined by the evenly distributed residuals in the regression line. The variance inflation factor (VIF) values below 10.0 were used to define the absence of multicollinearity. Additionally, the autocorrelation of the variables was verified by the Durbin–Watson test, and the values between 1.5 and 2.5 showed that there was no autocorrelation in the data. Statistical significance was set at 5%. 

## 3. Results 

### Characteristics of Subjects

**Figure 1:**
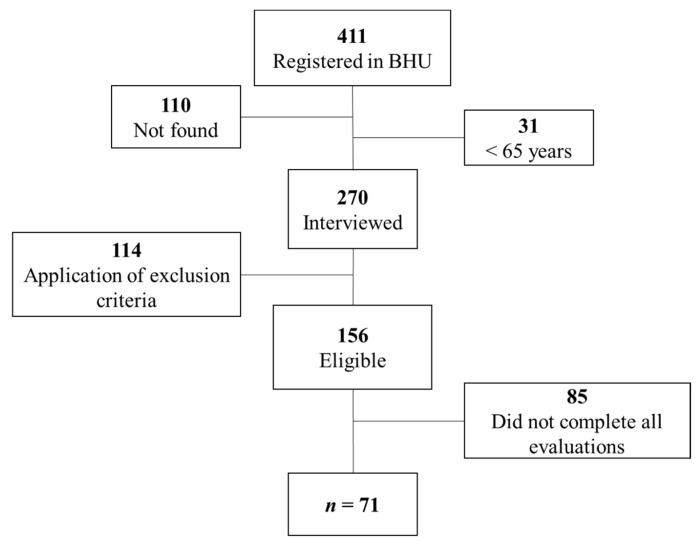
Sample recruitment flowchart. Abbreviations: BHU: basic health units.

Four hundred and eleven elderly women were recruited based on their registration at the BHU. Of these, one hundred and ten addresses were not located, and thirty-one were not age-appropriate. Two hundred and seventy elderly women were interviewed in their homes, and one hundred and fourteen did not meet the inclusion criteria. One hundred and fifty-six older community-dwelling women were eligible for the evaluation procedures. Eighty-five elderly women did not complete all assessments, while seventy-one completed all procedures (Figure 1).

Seventy-one older community-dwelling women (75 ± 7 years old) participated in the study. The characteristics of body composition, muscle strength, and biomarker plasma levels are presented in Table 1.

The low ALM group presented lower values of trunk fat mass, total fat mass, and HGS. Furthermore, plasma levels of adiponectin, IL-8, and sTNFr1 were higher in the low ALM group compared to the normal ALM group (*p* < 0.05) (Table 2).

There was a significant positive correlation between ALM and trunk fat mass (r = 0.78; *p* < 0.01) and a significant negative correlation between ALM and adiponectin (r = −0.26; *p* = 0.03), IL-8 (r = −0.26; *p* = 0.03), and sTNFr-1 (r = −0.25; *p* = 0.04) (Table 3). In univariate regression analyses, having ALM as a dependent variable revealed that the blood adiponectin, sTNFr1, and IL-8 levels were negatively associated with ALM in older women. In multivariate analyses, including trunk fat mass and biomarkers as independent variables, the trunk fat mass and adiponectin composed the model that better explained the ALM. Therefore, in clinical terms, total trunk fat and adiponectin predicted ALM in community-dwelling older women. Overall, these outcomes explained 65% (β = 0.76; adjusted R^2^ = 0.65; *p* = 0.001) of the ALM, and adiponectin explained 14% of the ALM variability (β = −0.39; R^2^ = −0.14; *p* < 0.01) of community-dwelling older women (Table 3).

## 4. Discussion

To our knowledge, this is the first study to assess the association between appendicular muscle mass using DXA and a broad panel of blood biomarkers related to sarcopenia. In our screening, low appendicular muscle mass or the risk of sarcopenia was present in 69% of the community-dwelling older women. Handgrip strength (HGS), total fat mass, and trunk fat mass were significantly lower in the low ALM group. Regarding biomarkers, plasma levels of adiponectin, sTNFr1, and IL-8 were higher in the low ALM group and demonstrated an association with adiponectin, sTNFr1, and IL-8 with ALM. In clinical terms, community-dwelling older women with less muscle mass had higher blood levels of these biomarkers (Table 2). Of note, our post-hoc analysis from linear multiple regression (correlation coefficient of 0.26 between ALM and adiponectin) revealed a large effect size of 0.35 and a power of 0.85 [14].

Noteworthy, older women with low ALM also presented low HGS, as in cases of confirmed sarcopenia [1,2,14,15]. There is still debate concerning the relationship between biomarkers and sarcopenia [4,5,7,8,10,16,17]. This is because once a skeletal muscle has become a secretory organ, inflammatory and muscle cells can work together to produce the myokines that induce sarcopenia [5,18,19].

There is growing evidence suggesting that chronic low-grade inflammation, or inflammation, could play a key role in the development of sarcopenia [4,5,7,10,15,16,17,18]. Thus, high blood level of proinflammatory biomarkers, including IL-6, IL-8, and IL-15, can affect skeletal muscle mass and are suggestive of inflammation, whereas high blood level of anti-inflammatory biomarkers, i.e., IL-4, IL-10, and IL-15 can counteract the generation and activity of proinflammatory cytokines and consequently the muscle atrophy and sarcopenia [5,10,17,18,19,20,21,22].

Among all evaluated biomarkers, adiponectin, IL-8, and sTNFr1 correlated negatively with ALM (Table 3). It is unclear whether IL-8, a chemotactic factor that leads to inflammation, contributes to sarcopenia [10,23]. As seen in cachexia and frailty, higher levels of IL-8 indicate a more prepared and active innate immune response [24]. The findings of the present study reinforce a previous work, including older people from the United Kingdom, which demonstrated the effect between higher levels of IL-8, and lower ALM, i.e., subjects with higher risk of sarcopenia [25].

Thus, the findings of the study are consistent with studies that found an increase in sTNFr1 blood levels to be negatively linked to muscle mass parameters [26]. Furthermore, over a five-year period, computed tomography as well as the Jamar dynamometer revealed a significant link between increased sTNFr1 blood levels and loss of muscle mass and strength [26]. However, Lustosa et al. (2017) found greater sTNFr1 blood levels in older individuals who were not sarcopenic [27]. In this sense, as the connection between sTNFr1 and sarcopenia or low muscle mass remains unclear, further research is needed.

Sarcopenia is linked to the adipokines, i.e., leptin and adiponectin [16], which are secreted by adipose and musculoskeletal tissues [10]. Currently, it is unclear whether leptin and muscle mass or sarcopenia interact [5,10,28]. As far as we know, leptin blood levels have proinflammatory effects [29]. The study by Li et al. (2019), which included older sarcopenic and nonsarcopenic individuals, showed that sarcopenic individuals had considerably higher leptin blood levels, which were linked to the severity and risk of sarcopenia [30]. In accordance, Kohara et al. (2011) observed that high leptin blood levels are greater in individuals with sarcopenia and visceral obesity than in individuals with only one condition, suggesting that leptin blood levels are associated with sarcopenia, independent of visceral fat [31]. Although leptin blood levels were lower in the low ALM group in our sample, this difference was not statistically significant.

A biomarker with antidiabetic, anti-inflammatory, and antiatherogenic properties is adiponectin [8,16,32]. It is produced by skeletal muscle and adipose tissue, has metabolic effects primarily in the liver and skeletal muscle, and modulates inflammatory processes by preventing the production of proinflammatory markers, including IL-6, IL-18, and TNF- [16,32,33]. A positive muscle function regulator also directly produces injured fibers and triggers the metabolism of muscle cells [8,9,31,32,33].

According to a meta-analysis, including seven studies, those with sarcopenia (*n* = 557) were more likely to have higher levels of adiponectin [8]. In addition, the prevalence of sarcopenia was reported to differ between men and women, and meta-regression analysis revealed an important role for females in explaining the association between sarcopenia and adiponectin, suggesting that women have higher levels of adiponectin than men, as they seem to express more plasma adiponectin than men, regardless of fat mass and BMI, due to the influence of sex hormones [8]. However, adiponectin blood levels in studies using DXA did not differ significantly between subgroups in analysis [8]. In another line, our findings are consistent with the results of Rossi et al. (2019), who compared the inflammatory profile of the Brazilian seniors and observed that the sarcopenic group had a higher concentration of adiponectin [16]. Although increased adiponectin levels in sarcopenia are still being studied [4,5,8,10,16], some possible mechanisms have been proposed to explain the peculiarity: down-regulation of adiponectin receptor signaling [8,32], deposition of adipose tissue in muscles that may influence adiponectin expression [8,34], and activation of catabolism related to the presence of other comorbidities [8,33]. Of note, adiponectin blood level was significantly higher in the low ALM group in our sample and was inversely associated with ALM (r = −0.259; *p* = 0.02) explaining 14% of the ALM variability (β = −0.39; R^2^ = −0.14; *p* < 0.00), occurring independently of the total fat mass or trunk fat mass.

Therapeutic targets for the treatment of sarcopenia might include anti-inflammatory cytokines [5,33]. It is generally agreed upon that regulating IL-6 levels may be a therapeutic approach to keep skeletal muscle healthy [5,33,35]. Considering the evidence of adiponectin’s anti-inflammatory and regenerative role, our findings point to it as a potential biomarker linked to muscle damage brought on by low ALM and encourage more study into diagnostics and therapy.

The development of chronic inflammation and sarcopenia may be influenced by adipose tissue, which can also lead to sarcopenia and sarcopenic obesity [10,16,35]. As a result, obese people who have more visceral subcutaneous adipose tissue are more likely to have greater levels of adiponectin, indicating that the distribution of adipose tissue also influences the release of adipokines [10,34]. Thus, understanding that body fat, especially trunk fat, is metabolically active and secretes inflammatory biomarkers, including adiponectin [34], it is important to highlight that the best model that explained the low appendicular muscle mass was adiponectin and the trunk fat mass (Table 3) in older community-dwelling women. In addition, to clarify the relationship between ALM, fat mass, and adiponectin, we evaluated additional models using adiponectin as a dependent variable, including total fat mass, total lean mass, and ALM as independent variables. Therefore, we found that ALM was the only significant predictor [F (1, 69) = 12.79; *p* < 0.01; R^2^ = 0.14; β = −0.39], explaining 14% of adiponectin, reinforcing the association between muscle mass and adiponectin independently of body fat.

When the model of the analysis was adjusted for age, the results remained the same (R = 0.80; adjusted R^2^ = 0.63; *p* < 0.001), suggesting that trunk fat mass and adiponectin are possible predictors of appendicular muscle mass in older community-dwelling women. Thus, our findings together allow us to speculate on the involvement of adiponectin in the modulation of inflammatory control and ALM, influencing positively the balance between the anti-inflammatory (IFN, IL-4, IL-5, and IL-10) and proinflammatory biomarkers (IL-2, IL-6, sTNFr2, and TNF) in community-dwelling older women. However, the biomarkers were dosed systemically in our group, but possibly future research that measures the biomarkers directly in the muscle would provide more illuminating results. More research advances are needed to explore whether there is a cause-effect relationship in the interaction between muscle and biomarkers.

Notably, there was no difference in the clinical, sociodemographic, cultural, or functional variables between the older, normal, and low ALM groups, showing there was no data interpretation bias. The present study’s stronger points were its methodological rigor, use of one of the most recommended methods to measure body composition, participants fasting prior to body composition and blood level assessments, the blinding of the evaluators to the DXA and strength test, as well as the rigor in the exclusion criteria used.

The contribution of the present study is to show that high levels of adiponectin are associated with lower appendicular muscle mass in community-dwelling elderly women. The practical implications of this study are as follows: (1) Older women with lower ALM assessed by DXA also have lower HGS. Low levels of these clinical features are directly associated with sarcopenia in older women; (2) higher adiponectin levels were associated with lower appendicular muscle mass among community-dwelling older women; (3) contribution to clinical or public health strategies aimed at early assessment and diagnosis of sarcopenia and other diseases related to low muscle mass in old women. The limitation of this study was the short observation time (cross-sectional study); only older community-dwelling women were investigated; our findings should not be extrapolated to the population of clinical settings, long-term care facilities, or hospital environments; and we did not assess a cutoff point for adiponectin to predict ALM. Future studies that determine a cutoff point for adiponectin based on ALM in both sexes may further enhance our understanding of the association of these variables with sarcopenia.

## 5. Conclusions

High adiponectin levels in the blood contribute to lower ALM in community-dwelling older women. Therefore, initiatives for the diagnosis and treatment of sarcopenia in community-dwelling older women may focus on this adipokine as a possible biomarker of sarcopenia.

## Figures and Tables

**Table 1:** Characteristics of older community-dwelling women. (*n* = 71).

	Mean (± SD)
Age (years)	75 (7)
Appendicular lean mass (kg)	14.38 (2.63)
Trunk fat mass (kg)	12.44 (4.29)
Total fat mass (kg)	23.01 (6.82)
HGS (kgf)	19.9 (6.38)
Adiponectin (µg/mL)	49.34 (6.94)
BDNF (µg/mL)	2.51 (0.93)
IFN (ng/mL)	1.56 (1.52)
IL 2 (ng/mL)	5.13 (8.51)
IL 4 (ng/mL)	2.39 (3.10)
IL 5 (ng/mL)	1.05 (1.21)
IL 6 (ng/mL)	17.45 (4.23)
IL 8 (ng/mL)	23.73 (9.03)
IL10 (ng/mL)	2.13 (3.40)
Leptin (µg/mL)	1.84 (0.27)
Resistin (µg/mL)	1.62 (0.35)
sTNFr1 (µg/mL)	3.93 (3.29)
sTNFr2 (µg/mL)	2.11 (0.48)
TNF (ng/mL)	1.31 (1.87)

Data presented as mean ± standard deviation (SD). Abbreviations: BDNF: brain-derived neurotrophic factor; HGS: handgrip strength; sTNFr1: soluble tumor necrosis factor receptor 1; sTNFr2: soluble tumor necrosis factor receptor 2; IFN: interferon; TNF: tumor necrosis factor; IL-10: interleukin-10; IL-5: interleukin-5; IL-4: interleukin-4; IL-2: interleukin-2. IL-6: interleukin-6; IL-8: interleukin-8.

**Table 2:** Anthropometric variables, muscle strength, and biomarker blood levels stratified according to the appendicular lean mass in older community-dwelling women. (*n* = 71).

	Normal ALM (*n* = 22)	Low ALM (*n* = 49)	*p*-Value
Age (years)	73 (7)	75 (7)	0.19
ALM (kg)	17.49 (1.41)	12.98 (1.66)	<0.01 *
Trunk fat mass (kg)	16.73 (2.67)	10.52 (3.40)	<0.01 *
Total fat mass (kg)	29.87 (5.08)	19.92 (5.02)	<0.01 *
HGS (kgf)	25.38 (5.74)	17.47 (5.02)	<0.01 *
Adiponectin (µg/mL)	46.11 (9.61)	50.79 (4.78)	0.01 *
BDNF (µg/mL)	2.27 (0.83)	2.62 (0.97)	0.12
IFN (ng/mL)	1.46 (0.34)	1.61 (1.82)	0.25
IL-2 (ng/mL)	4.12 (0.39)	5.58 (10.25)	0.89
IL-4 (ng/mL)	1.98 (0.17)	2.57 (3.73)	0.21
IL-5 (ng/mL)	0.74 (0.16)	1.20 (1.44)	0.17
IL-6 (ng/mL)	16.58 (2.81)	17.84 (4.70)	0.32
IL-8 (ng/mL)	20.71 (4.75)	25.08 (10.15)	0.01 *
IL-10 (ng/mL)	1.59 (0.30)	2.37 (4.08)	0.46
Leptin (µg/mL)	1.90 (0.20)	1.81 (0.29)	0.47
Resistin (µg/mL)	1.59 (0.34)	1.64 (0.36)	0.55
sTNFr1 (µg/mL)	2.74 (2.62)	4.46 (3.45)	0.03 *
sTNFr2 (µg/mL)	1.98 (0.41)	2.17 (0.51)	0.23
TNF (ng/mL)	1.06 (0.17)	1.43 (2.25)	0.14

Data presented as mean ± standard deviation (SD). Abbreviations: ALM = appendicular lean mass; BDNF = brain-derived neurotrophic factor; HGS: handgrip strength; Stnfr1 = soluble tumor necrosis factor receptor 1; STNFr2 = soluble tumor necrosis factor receptor 2; IFN = interferon; TNF = tumor necrosis factor; IL-10 = interleukin-10; IL-5 = interleukin-5; IL-4 = interleukin-4; IL-2 = interleukin-2. IL-6 = interleukin-6; IL-8 = interleukin-8. Note: * *p*-value < 0.05.

**Table 3:** Independent contributors to appendicular lean mass in community-dwelling older women (*n* = 71).

Independent Variables	*r*	Univariate		Multivariate			*p*-Value
		R^2^ Adjusted	β	*p*-Value	R^2^ Adjusted	β	
**Trunk fat mass**	0.78 ± 0.01	0.58	0.76	<0.01 *	0.65	0.76	0.001 *
**Adiponectin**	−0.26 ± 0.03	0.14	−0.39	0.001 *			
IL-8	−0.26 ± 0.03	0.03	−0.22	0.06			NS
sTNFr-1	−0.25 ± 0.04	0.05	−0.26	0.03 *			NS

Β: beta coefficient; *r*: coefficient of Spearman correlation; R2 adjusted: adjusted coefficient of determination. BDNF: brain-derived neurotrophic factor; STNFR1: soluble tumor necrosis factor receptor 1; IL-8: interleukin-8. NS: non significance. Note: * *p*-value < 0.05.

## Data Availability

The data presented in this study are available upon request from the corresponding author. The data are not publicly available due to the privacy guarantee of the data collected individually.

## References

[1] Baumgartner R.N., Koehler K.M., Gallagher D., Romero L., Heymsfield S.B., Ross R.R., Garry P.J., Lindeman R.D. (1998). Epidemiology of Sarcopenia among the Elderly in New Mexico. Am. J. Epidemiol..

[2] Cruz-Jentoft A.J., Bahat G., Bauer J., Boirie Y., Bruyère O., Cederholm T., Cooper C., Landi F., Rolland Y., Sayer A.A. (2019). Sarcopenia: Revised European consensus on definition and diagnosis. Age Ageing.

[3] de Souza L.F., Fontanela L.C., Gonçalves C., Mendrano A.L., Freitas M.A., Danielewicz A.L., de Avelar N.C.P. (2021). Cognitive and behavioral factors associated to probable sarcopenia in community-dwelling older adults. Exp. Aging Res..

[4] Curcio F., Ferro G., Basile C., Liguori I., Parrella P., Pirozzi F., Della-Morte D., Gargiulo G., Testa G., Tocchetti C.G. (2016). Biomarkers in sarcopenia: A multifactorial approach. Exp. Gerontol..

[5] Pan L., Xie W., Fu X., Lu W., Jin H., Lai J., Zhang A., Yu Y., Li Y., Xiao W. (2021). Inflammation and sarcopenia: A focus on circulating inflammatory cytokines. Exp. Gerontol..

[6] Arrieiro A.N., Soares L.A., Prates A.C.N., Figueiredo P.H.S., Costa H.S., Simão A.P., Neves C.D.C., dos Santos J.M., Santos L.M.D.M., Avelar N.C.P. (2021). Inflammation Biomarkers Are Independent Contributors to Functional Performance in Chronic Conditions: An Exploratory Study. Int. J. Med. Sci. Health Res..

[7] Argilés J.M., Campos N., Lopez-Pedrosa J.M., Rueda R., Rodriguez-Mañas L. (2016). Skeletal Muscle Regulates Metabolism via Interorgan Crosstalk: Roles in Health and Disease. J. Am. Med. Dir. Assoc..

[8] Komici K., Iacono A.D., De Luca A., Perrotta F., Bencivenga L., Rengo G., Rocca A., Guerra G. (2021). Adiponectin and Sarcopenia: A Systematic Review with Meta-Analysis. Front. Endocrinol..

[9] Fiaschi T., Tedesco F.S., Giannoni E., Diaz-Manera J., Parri M., Cossu G., Chiarugi P. (2010). Globular Adiponectin as a Complete Mesoangioblast Regulator: Role in Proliferation, Survival, Motility, and Skeletal Muscle Differentiation. Mol. Biol. Cell.

[10] Wang T. (2022). Searching for the link between inflammaging and sarcopenia. Ageing Res. Rev..

[11] Neves C.D., Lage V.K., Lima L.P., Matos M.A., Vieira L., Teixeira A.L., Figueiredo P.H., Costa H.S., Lacerda A.C.R., Mendonça V.A. (2021). Inflammatory and oxidative biomarkers as determinants of functional capacity in patients with COPD assessed by 6-min walk test-derived outcomes. Exp. Gerontol..

[12] Dias J.A., Ovando A.C., Külkamp W., Junior N.G.B. (2010). Hand grip strength: Evaluation methods and factors influencing this measure. Rev. Bras. De Cineantropometria E Desempenho Hum..

[13] Studenski S.A., Peters K.W., Alley D.E., Cawthon P.M., McLean R.R., Harris T.B., Ferrucci L., Guralnik J.M., Fragala M.S., Kenny A.M. (2014). The FNIH Sarcopenia Project: Rationale, Study Description, Conference Recommendations, and Final Estimates. J. Gerontol. A Biol. Sci. Med. Sci..

[14] Can B., Kara O., Kizilarslanoglu M.C., Arik G., Aycicek G.S., Sumer F., Civelek R., Demirtas C., Ulger Z. (2016). Serum markers of inflammation and oxidative stress in sarcopenia. Aging Clin. Exp. Res..

[15] Rossi F.E., Lira F.S., Silva B.S.A., Freire A.P.C.F., Ramos E.M.C., Gobbo L.A. (2018). Influence of skeletal muscle mass and fat mass on the metabolic and inflammatory profile in sarcopenic and non-sarcopenic overfat elderly. Aging.

[16] Parentoni A.N., Lustosa L.P., Dos Santos K.D., Sá L.F., Ferreira F.O., Mendonça V.A. (2013). Comparação da força muscular respiratória entre os subgrupos de fragilidade em idosas da comunidade. Fisioter. E Pesqui..

[17] Peake J.M., Della Gatta P., Suzuki K., Nieman D.C. (2015). Cytokine expression and secretion by skeletal muscle cells: Regulatory mechanisms and exercise effects. Exerc. Immunol. Rev..

[18] Pedersen B.K., Febbraio M.A. (2008). Muscle as an Endocrine Organ: Focus on Muscle-Derived Interleukin-6. Physiol. Rev..

[19] Pedersen B.K., Fischer C. (2007). Physiological roles of muscle-derived interleukin-6 in response to exercise. Curr. Opin. Clin. Nutr. Metab. Care.

[20] Hofmann S., Rösen-Wolff A., Tsokos G., Hedrich C. (2012). Biological properties and regulation of IL-10 related cytokines and their contribution to autoimmune disease and tissue injury. Clin. Immunol..

[21] Heredia J.E., Mukundan L., Chen F.M., Mueller A.A., Deo R.C., Locksley R.M., Rando T.A., Chawla A. (2013). Type 2 Innate Signals Stimulate Fibro/Adipogenic Progenitors to Facilitate Muscle Regeneration. Cell.

[22] Harada A., Sekido N., Akahoshi T., Wada T., Mukaida N., Matsushima K. (1994). Essential involvement of interleukin-8 (IL-8) in acute inflammation. J. Leukoc. Biol..

[23] Wilson D., Jackson T., Sapey E., Lord J.M. (2017). Frailty and sarcopenia: The potential role of an aged immune system. Ageing Res. Rev..

[24] Westbury L.D., Fuggle N.R., Syddall H.E., Duggal N.A., Shaw S.C., Maslin K., Dennison E.M., Lord J.M., Cooper C. (2017). Relationships Between Markers of Inflammation and Muscle Mass, Strength and Function: Findings from the Hertfordshire Cohort Study. Calcif. Tissue Res..

[25] Singh T., Newman A.B. (2011). Inflammatory markers in population studies of aging. Ageing Res. Rev..

[26] Schaap L.A., Pluijm S.M.F., Deeg D.J.H., Harris T.B., Kritchevsky S., Newman A.B., Colbert L.H., Pahor M., Rubin S.M., Tylavsky F.A. (2009). Higher Inflammatory Marker Levels in Older Persons: Associations With 5-Year Change in Muscle Mass and Muscle Strength. J. Gerontol. Ser. A.

[27] Lustosa L.P., Batista P.P., Pereira D.S., Pereira L.S.M., Scianni A., Ribeiro-Samora G.A. (2017). Comparison between parameters of muscle performance and inflammatory biomarkers of non-sarcopenic and sarcopenic elderly women. Clin. Interv. Aging.

[28] Abella V., Scotece M., Conde J., Pino J., Gonzalez-Gay M.A., Gómez-Reino J.J., Mera A., Lago F., Gómez R., Gualillo O. (2017). Leptin in the interplay of inflammation, metabolism and immune system disorders. Nat. Rev. Rheumatol..

[29] Li C.-W., Yu K., Shyh-Chang N., Li G.-X., Jiang L.-J., Yu S.-L., Xu L.-Y., Liu R.-J., Guo Z.-J., Xie H.-Y. (2019). Circulating factors associated with sarcopenia during ageing and after intensive lifestyle intervention. J. Cachexia-Sarcopenia Muscle.

[30] Kohara K., Ochi M., Tabara Y., Nagai T., Igase M., Miki T. (2011). Leptin in Sarcopenic Visceral Obesity: Possible Link between Adipocytes and Myocytes. PLoS ONE.

[31] Fiaschi T., Cirelli D., Comito G., Gelmini S., Ramponi G., Serio M., Chiarugi P. (2009). Globular adiponectin induces differentiation and fusion of skeletal muscle cells. Cell Res..

[32] Wolf A.M., Wolf D., Rumpold H., Enrich B., Tilg H. (2004). Adiponectin induces the anti-inflammatory cytokines IL-10 and IL-1RA in human leukocytes. Biochem. Biophys. Res. Commun..

[33] Belizário J.E., Fontes-Oliveira C.C., Borges J.P., Kashiabara J.A., Vannier E. (2016). Skeletal muscle wasting and renewal: A pivotal role of myokine IL-6. SpringerPlus.

[34] Guenther M., James R., Marks J., Zhao S., Szabo A., Kidambi S. (2014). Adiposity distribution influences circulating adiponectin levels. Transl. Res..

[35] Sell H., Habich C., Eckel J. (2012). Adaptive immunity in obesity and insulin resistance. Nat. Rev. Endocrinol..

